# Comparison of one-level microendoscopy laminoforaminotomy and cervical arthroplasty in cervical spondylotic radiculopathy: a minimum 2-year follow-up study

**DOI:** 10.1186/1749-799X-8-48

**Published:** 2013-12-17

**Authors:** Guo Min Liu, Ya Jun Wang, Dong Sheng Wang, Qin-Yi Liu

**Affiliations:** 1 Department of Orthopedic Surgery, the Second Hospital of Jilin University, Changchun 130041, People's Republic of China

**Keywords:** Microendoscopy, Laminoforaminotomy, Arthroplasty, Cervical spondylotic radiculopathy

## Abstract

**Background:**

This study aims to compare the perioperative parameters and clinical results between microendoscopy laminoforaminotomy (MELF) and cervical arthroplasty (CA) in the treatment of one-level cervical spondylotic radiculopathy in a retrospective study.

**Methods:**

From 2003 to 2007, a total of 97 patients with one-level cervical spondylotic radiculopathy were treated. Forty-five patients underwent CA. Fifty-two patients underwent MELF. Patient demographics and operative data were collected with a minimum 2-year follow-up. Perioperative parameters were compared. Clinical assessment in terms of neck disability index (NDI), short form (SF)-36, and visual analogue scale (VAS) of arm pain and neck pain was performed prior to surgery and at 1.5, 3, 6, 12, and 24 months after surgery.

**Results:**

Fluoroscopy time (CA, 60.3 s; MELF, 12.1 s; *P* < 0.01) and surgical time (CA, 95.1 min; MELF, 24.0 min; *P* < 0.01) were significantly longer in the CA cases. Shorter hospitalized days (CA, 1.1 days; MELF, 0.13 days; *P* < 0.01) and less estimated blood loss (EBL; CA, 75.8 ml; MELF, 31.9 ml; *P* < 0.01) were observed in the MELF group. Both CA and MELF groups showed significant improvement in NDI, VAS of neck pain and arm pain, and SF-36 (*P* < 0.05 for each) at 1.5, 3, 6, 12, and 24 months after surgery, but there was no significant difference between them (*P* > 0.05).

**Conclusions:**

As alternatives of anterior cervical decompression and fusion (ACDF), both CA and MELF can produce satisfactory clinical outcomes. MELF has the additional benefits of less blood loss, less surgical time, less X-ray time, and shorter hospital stay.

## Introduction

Cervical spondylotic radiculopathy can be treated by either anterior or posterior approach. The posterior approach has limitations in dealing with central disc herniation or spondylosis. In addition, the open posterior approach is associated with significant muscle spasm that has close relation to post-surgery neck pain. Subsequently, anterior cervical discectomy and fusion (ACDF) became popular in treating degenerative cervical disc disease. Compared with the open posterior approach, the anterior approach is generally associated with shorter recovery time and less neck muscular spasm morbidity, but has a greater potential for complications. Longer follow-up has presented that up to 25% of patients may develop recurrent radicular symptoms from adjacent segment degeneration 10 years after ACDF [1,2].

Cervical arthroplasty (CA) has been introduced as a new alternative to ACDF to treat cervical spondylotic radiculopathy and myelopathy. In theory, CA has the advantage of motion maintenance of the affected segment, which might decrease the likelihood of adjacent segmental disease [3-5]. Multicenter clinical trials have shown that arthroplasty is an effective treatment method for cervical radiculopathy and myelopathy [6,7].

Progress in minimally invasive spinal technique makes it possible to treat cervical spondylotic radiculopathy through a posterior working tube. Microendoscopic laminoforaminotomy (MELF) allows for motion preservation via a minimally invasive approach. Such an approach shares the advantage of maintenance of motion with arthroplasty, but without the need for instrumentation. Furthermore, this technique has a potential benefit for neck muscle with a muscle splitting technique, which may minimize hospitalization and recovery time as well as decrease the axial neck pain [8-10]. The purpose of this study was to compare the perioperative parameters and clinical outcomes between CA and MELF in treating one-level cervical spondylotic radiculopathy.

## Patients and methods

### Patients

From 2003 to 2007, a total of consecutive 45 patients treated with CA and 52 patients treated with MELF were assessed at the Carolina Neurosurgery & Spine Associates. Inclusion criteria were as follows: (1) for clinical manifestations, shoulder and neck pain extends down according to the innervation to the forearms and fingers; (2) for specialist examination, the upper arm may be hypoesthetic, and the tendon reflexes weaken. The Spurling signs are positive. (3) For imaging examination, MRI shows a single intervertebral disc hernia. The selection of surgical method, CA or MELF, was randomized according to the intention of patients. Two chief surgeons and six deputy surgeons were involved in the research. The minimum follow-up was 24 months. Preoperatively, patient demographic data were collected and compared for age, gender, weight, and height. Arm pain and neck pain were quantified by visual analogue scale (VAS). Functional evaluation was performed using the neck disability index (NDI) and Short Form-36 (SF-36). Meanwhile, the operative levels were also compared before the surgery. For perioperative parameters, operation time, X-ray time, total amount of blood loss, length of hospital stay were compared (Table 1). The 45 patients treated with CA included our learning cases, the 52 patients treated with MELF were from our established experience. Clinical assessment in terms of NDI, VAS, and SF-36 was performed at 1.5, 3, 6, 12, and 24 months after surgery. This study was approved by the Jilin University Second Hospital Ethical Committee (no. 2012060). Experimental research was in compliance with the Helsinki Declaration.

**Table 1:** Preoperative patient demographics

	**CA**	**MELF**
Total patients enrolled	45	52
Female	26 (57.8%)	18 (34.6%)
Male	19 (42.2%)	34 (65.4%)
Age (years)	47.6 ± 7.9 (range 31–66)	43.0 ± 10.0 (range 24–62)
Physical characteristics		
Height (in.)	67.7 ± 3.9	68.3 ± 4.6
Weight (lb)	172.5 ± 40.0	188.1 ± 42.7
Symptom scores		
Arm pain VAS	69.1 ± 13.7	67.3 ± 13.3
Neck pain VAS	68.7 ± 11.3	66.2 ± 12.4
NDI	45.9 ± 10.1	45.5 ± 13.0
SF-36	34.3 ± 5.8	35.1 ± 5.3
Surgery level		
C3/4	0	0
C4/5	2 (4.4%)	2 (3.9%)
C5/6	26 (57.8%)	15 (28.8%)
C6/7	17 (37.8%)	30 (57.7%)
C7/T1	0	5 (9.6%)

Data are stated as mean ± standard deviation or number (percentage).

### Technique for MELF

General endotracheal anesthetization was performed, and patient was positioned with Mayfield head holder in a sitting position. Fluoroscopy was used, and a spinal needle was initially placed alongside the neck to make sure correct level prior to skin incision. When the level was identified, the needle was inserted into the skin 2 cm off the midline and directed to the back of the cephalad lamina of the target level. Subsequently, the needle was removed, and a 16-mm obliquely (parallel to dermatoglyph) angled incision was made around the centered puncture point. Once the first dilator was in the right place, the K-wire was removed, and a subperiosteal dissection off the lamina and facet was performed. The facet and lamina 'step off’ could also be identified by palpation and confirmed with fluoroscopy and subsequent endoscopy. Followed with other dilators placed over each other, the former dilator was maintained with some constant pressure to ensure that the system did not migrate when each dilator was inserted. Finally, the operative cylinder was placed, and the dilators removed. The cylinder was caudally angled to allow any bleeding out of the operative field. The microendoscope was then anchored in the cylinder. To identify step off once more was very important. Ideally, the cylinder was right centered at the disc space in the cephalad-caudal direction, and the target pedicle was centered medial to lateral in the surgery field.

The lateral lamina and medial facet were burred with a high-speed electric drill to access the targeted foramen. Small Kerrisons were used to complete the foraminotomy, ensuring nerve root exposure from its origin at the thecal sac laterally across the cephalad edge of the pedicle.

When the foraminotomy was finished, the floor of the canal and foramen were felt for soft disc herniations. This was safely completed by inserting a nerve hook along the medial edge of the pedicle to the floor of the canal and then carefully rotated medially and then cephalad over the disc space as it was rotated out of the foramen. Disc extrusions might be mobilized in this way and removed with pituitary rongeurs. Contained herniation may require the use of a micro down-angled curette to open the remaining ligament or annulus fibers prior to mobilizing the fragments. Once the foraminotomy and discectomy were accomplished, the pulse of the nerve root and dural sac could usually be seen. The wound was then irrigated and closed in the customary fashion.

### Technique for anterior CA

General endotracheal anesthetization was performed, and patient was positioned supine on a radiolucent table. The anterior part of the cervical spine was exposed with a standard Smith-Robinson approach. Fluoroscopy was used, and a spinal needle was placed alongside the neck to ensure correct level. The longus colli was elevated through the uncinate process and over the foramen transversarium. Anterior osteophytes were removed with a burr or a Leksell rongeur and then the anterior margin of the disc space was flushed with the rest of the vertebral body. The anterior annulus was incised with a number-15 scalpel; pituitary rongeurs and small curettes were used to remove the initial disc material and fragments. When the initial discectomy had been performed, the lateral borders of the uncinate processes were carefully identified. At this point, both end plate preparation and canal/nerve decompression were performed. When the pins had been inserted and distraction had been applied, the burr was then used to bur and flatten the end plates. A thorough decompression could be achieved by burring all posterior vertebral and uncinate osteophytes. Then, the sizing trials were used to determine the appropriate size of the arthroplasty device. The trial prosthesis should fit snugly within the disc space. The selected device was then inserted into the interspace under the fluoroscopic guidance. If required, screws were placed with the use of standard techniques. The wound was then irrigated and closed in the customary fashion.

### Statistical analysis

Data were stated as mean ± standard deviation (SD) for continuous variables and as number (or percentage) for counting variables. Statistical significance was tested using Student *t* test for continuous variables and Pearson *χ*^2^ test for counting variables. *P* < 0.01 indicated statistically significant difference. Perioperative parameters (surgical time, EBL, X-ray exposure, and hospital stay) were compared between CA and MELF after a 2-year follow-up. All analyses were done using SPSS 13.0 software (SPSS Inc., Chicago, IL, USA).

## Results

The mean age for CA and MELF was 47.6 and 43.0 years, respectively. There was a higher female patient proportion in the MELF group (65.4%) than in the CA group (42.2%). However, preoperative NDI, VAS, and SF-36 results were not significantly different between the two groups (*P* > 0.05 in Student *t* test for each). Some difference appeared in the level of surgery. The MELF group included less C5/6, more C6/7, and particular five C7/T1 patients (Table 1).

The average follow-up was 28.6 months with a range of 24–60 months. None of the patients in the MELF group needed open surgery. The statistical evaluation showed a significant difference in the operative time, fluoroscopy time, EBL, and hospital stay (*P <* 0.01 in Student *t* test for each). The average operation time was 95.1 min in the CA group, while 24.0 min in the MELF group (*P <* 0.01). Estimated blood loss (EBL) in the MELF group (31.9 ml) was less than that in the CA group (75.8 ml) (*P <* 0.01). None of these two groups of patients required blood transfusions. The length of hospital stay of the MELF group was shorter (0.13 days) than that of the CA group (1.1 days) (*P <* 0.01). The CA group needed more fluoroscopy time (60.3 s) than the MELF group (12.1 s) (*P <* 0.01) (see Table 2).

**Table 2:** Perioperative parameters (mean ± standard deviation, CA vs. MELF)

	**CA**	**MELF**
Surgical time (min)	95.1 ± 10.6	24.0 ± 5.4*
EBL (ml)	75.8 ± 15.7	31.8 ± 11.6*
X-ray exposure (s)	60.3 ± 8.5	12.1 ± 1.4*
Hospital stay (day)	1.1 ± 0.39	0.13 ± 0.13*

*P < 0.01 in Student *t* test indicates statistically significant difference compared with the CA group.

There were significant improvements postoperatively as compared with the preoperative status for both MELF and CA groups, based on the NDI score for functional disability and neurogenic symptoms, VAS for neck and arm pain, and SF-36 scores for physical component score (PCS) and mental component scores (MCS). However, no significant difference was observed between MELF and CA at 1.5, 3, and 6 months, and at 1- and 2-year follow-ups (Table 3). Considering that age, gender, weight, level of surgery, and mental factors may affect the clinical outcomes, subscale analyses were performed using Pearson *χ*^2^ test, based on VAS, NCI, and SF-36 scores. Gender and weight did not affect the outcomes within and between the CA and MELF groups (*P* > 0.05 for each). Age (subgroups 24–36, 37–50, and 51–66 years old) was associated with the postoperative recovery quality in the CA and MELF groups, and the younger has improved VAS and SF-36 (PCS and MCS) compared with the older (*P* < 0.01 for each). On the level of surgery, each of the C4/5, C5/6, and C6/7 subgroups did not affect the clinical outcomes when compared between CA and MELF. Meanwhile, the C6/7 surgery resulted in better VAS and SF-36 PCS in the CA and MELF groups, respectively (*P* < 0.01 for each). In addition, the MELF group rather than the CA group included five C7/T1 levels. On mental factor, higher SF-36 MCS was associated with higher SF-36 PCS and lower VAS (*P* < 0.01 for each).

**Table 3:** Follow-up parameters (mean ± standard deviation, CA vs. MELF)

	**Pre-op**	**1.5 months**	**3.0 months**	**6.0 months**	**12.0 months**	**24.0 months**
NDI						
CA	46.1 ± 7.1*	29.0 ± 5.5	23.1 ± 4.1	22.0 ± 3.5	21.3 ± 3.6	11.1 ± 2.5
MELF	45.1 ± 8.1*	31.0 ± 5.7	20.3 ± 4.1	20.1 ± 4.5	18.1 ± 3.1	10.2 ± 3.5
Neck pain VAS						
CA	70.4 ± 8.4*	31.6 ± 11.6	25.4 ± 7.2	23.6 ± 7.6	20.0 ± 6.5	18.1 ± 6.1
MELF	68.6 ± 10.4*	29.6 ± 7.6	24.4 ± 6.8	23.0 ± 7.4	21.1 ± 6.9	16.7 ± 6.4
Arm pain VAS						
CA	72.2 ± 7.4*	18.0 ± 5.2	19.1 ± 6.1	20.0 ± 4.5	18.1 ± 3.6	12.1 ± 2.7
MELF	71.3 ± 7.6*	18.0 ± 4.6	17.2 ± 5.8	16.4 ± 3.8	15.2 ± 3.4	12.2 ± 2.2
SF-36 PCS						
CA	33.3 ± 3.6*	40.0 ± 3.8	47.3 ± 4.6	48.1 ± 4.9	49.3 ± 5.6	52.2 ± 5.4
MELF	35.1 ± 4.6*	38.8 ± 4.4	44.7 ± 4.9	50.0 ± 5.5	49.1 ± 5.1	51.5 ± 5.2
SF-36 MCS						
CA	46.2 ± 3.6*	53.0 ± 4.4	55.1 ± 4.6	54.0 ± 5.4	55.3 ± 5.6	54.1 ± 5.3
MELF	47.1 ± 3.9*	55.3 ± 3.1	53.3 ± 4.3	53.4 ± 5.3	54.2 ± 5.3	54.3 ± 5.1

*P < 0.01 in Student *t* test indicates statistically significant difference compared with any other time point in the same group, based on the NDI score for functional disability and neurogenic symptoms, VAS for neck and arm pain, and SF-36 score for PCS and MCS. No significant difference was observed between MELF and CA at 1.5, 3, and 6 months, and at 1- and 2-year follow-ups (*P* > 0.05 in Student *t* test).

## Discussion

The ideal surgical treatment is to restore the original anatomic features and function. Such surgical treatment for cervical radiculopathy might be called 'functional spine surgery’ [11]. Recently, spine surgery has seen parallel interest and development in the fields of motion-preserving and minimally invasive surgery. If the spinal disease can be treated by minimally invasive surgery, it would be better to try avoiding use of instrument. When the original anatomy and function of the impaired spine cannot be salvaged, use of arthroplasty, rather than decompression and fusion treatments, is a good option. Both MELF and CA offer the practical advantage of preservation of segmental motion as well as the theoretical benefit of decreased adjacent level surgery [12-15]. These procedures would directly eliminate nerve-pinching pathological lesion with concomitant cosmetic benefits and shorter rehabilitation time [8,16].

Since its establishment in the 1950s, ACDF has been regarded as the classic method for degenerative cervical disc disease. Resection of the anterior cervical vertebrae or disc is required to gain access to compressive spondylotic lesions, most of which are disc diseases. At the same time, anterior discectomy will be followed by spinal reconstruction by bone graft fusion with or without instrumentation. ACDF procedure is easier and faster than open posterior laminoforaminotomy. The investigation of surgical outcomes of ACDF also showed high success rate immediately after surgery. However, during the process, the functional motion segment is destroyed and lost. The consequences of cervical fusion and subsequent loss of a motion of operative segment have raised more and more concern. DePalma et al. [17] reported on a series of 229 patients who underwent ACDF and found about 81% incidence of progressive adjacent segment disease. The adverse effects of the loss of motion segment might not lead to immediate disability in a short time, but loss of motion level will put more stress on adjacent levels which, subsequently, accelerates the degenerative process. Radiographic outcomes of adjacent segment degeneration have also been recently described in detail with time-point studies [15,18]. CA is designed to preserve normal spinal motion after anterior discectomy and avoid overstress and motion at adjacent segment which can protect adjacent levels from abnormal degeneration [19].

Posterior laminoforaminotomy has traditionally provided quick and durable relief of radiculopathy syndromes. However, the primary postoperative adverse complication is neck muscle spasm and neck pain related to the broad subperiosteal dissection of muscular insertions on the spinous process and lamina. In some patients, the pain and spasm are so severe that patients might feel stiffness at the neck, have to limit activity for weeks, and take more analgesics. The experience with MELF has demonstrated that adequate foraminal exposure and high rate of success for relief of radicular syndromes can be achieved with dilators technique. The use of 16-mm working tube and microendoscope allows very limited separation of muscles rather than cutting [20]. This surgical technique has the advantage of leaving the trapezius, splenius, and semispinalis muscles attached to the spinous process and lamina. This has been associated with very limited postoperative pain in most patients, which, in turn, reduces the need for postoperative narcotics and muscle relaxants and allows patients to return to full activity more quickly [10,21]. In addition, it is not necessary to cut too much facet joints because it can cause iatrogenic instability. As long as the facet joints can be kept more than 50%, there is little compromise of the sheer biomechanical strength of the cervical spine [22]. MELF also avoids the additional risks of injury to the anterior viscera of the neck, including the trachea, esophagus, carotid arteries, thymus, jugular veins, vagus nerve, recurrent laryngeal nerve, superior laryngeal nerve, and thoracic duct.

In this retrospective study, we compared the clinical outcomes and perioperative parameters of similar groups of patients treated with either CA or MELF. The preoperative information of both groups is similar, and both groups showed clinical improvement according to the NDI, VAS, and SF-36 form scores. No significant difference was observed between these two groups at a minimum of 24-month follow-up.

Theoretically, minimal surgery requires steep learning curve, longer operative time, and more fluoroscopy time than open surgery. However, in the present study, our results showed that MELF needed shorter fluoroscopy time during the surgery by about 12 s. The blood loss, operative time, and hospital stay time were also less than the CA group. The most important factors for surgeons to attain these aims are familiarity with the anatomy, a steric anatomy atlas at hand, and effort to decrease unnecessary interruption of peridural venous vessel to avoid excessive bleeding and coagulation time. Acquaintance of anatomy also makes surgeons easier to get to the affected area without too much unwanted fluoroscopic time.

There were five patients with C7/T1 radiculopathy treated by MELF. It is generally difficult for surgeons to treat C7/T1 herniation or osteophyte that causes foramen stenosis and radiculopathy. From the anterior approach, it is difficult to access the C7/T1 level, whereas posterior open laminoforaminotomy will cause unstable cervical-thoracic adjacent segments. Therefore, we performed decompression and laminoforaminotomy or/with discectomy through working tube and endoscope. Our patients had satisfactory clinical outcomes with MELF treatment.

In this study, we did not measure the motion of surgical level, adjacent level, and overall neck at the 24-month follow-up. Longer-term, multicenter studies will be required to definitively prove that segmental motion-preserving techniques such as CA and MELF statistically correlate with a lower incidence of adjacent segment disease and overall better clinical outcomes.

## Conclusions

Both CA and MELF have the advantage of preserving normal motion at an affected segment following decompression while striving to prevent compensatory motion and increasing intradiscal stress at adjacent motion segments that can accelerate the degeneration of adjacent levels. Improved imaging techniques for spinal image-guided systems and microendoscopic spinal equipment with proficiency of technique have made complex spine procedures simpler and safer. As alternatives of ACDF, both CA and MELF can produce satisfactory clinical outcomes. MELF may have the additional benefits of less blood loss, shorter surgical time, reduced X-ray exposure, and more cost-effective and shorter hospital stay.

## Abbreviations

ACDF: Anterior cervical decompression and fusion; EBL: Estimated blood loss; MCS: Mental component score; MELF: Microendoscopy laminoforaminotomy; MRI: Magnetic resonance imaging; NDI: Neck disability index; PCS: Physical component score; SF-36 form: Short form-36; VAS: Visual analogue scale.

## Competing interests

The authors declare that they have no competing interests.

## Authors' contributions

GML carried out the conception and design, acquisition and interpretation of data, and drafted the manuscript. YJW drafted the manuscript substantially. DSW carried out the acquisition and interpretation of data. QYL revised the manuscript critically for important intellectual content and gave final approval of the version to be published. All authors read and approved the final manuscript.
